# Contracting of private medical practitioners in a National Health Insurance pilot district: What has been the effect on primary healthcare utilisation indicators?

**DOI:** 10.4102/phcfm.v12i1.2563

**Published:** 2020-10-28

**Authors:** Hillary Mukudu, Kennedy Otwombe, Adam Fusheini, Jude Igumbor

**Affiliations:** 1 School of Public Health, Faculty of Health Sciences, University of the Witwatersrand, Johannesburg, South Africa; 2 Department of Statistics, Perinatal HIV Research Unit (PHRU), Faculty of Health Sciences, University of the Witwatersrand, Johannesburg, South Africa; 3 Department of Preventive and Social Medicine, University of Otago, Dunedin, New Zealand

**Keywords:** National Health Insurance pilot project, medical practitioners contracting

## Abstract

**Background:**

In 2012, the National Department of Health in South Africa started contracting of private medical practitioners (MPs) as part of the first phase of National Health Insurance (NHI) in 11 pilot districts to improve access to healthcare.

**Aim:**

The aim of this study was to describe the effect of contracting private MPs on the utilisation of primary healthcare (PHC) services in public healthcare facilities.

**Setting:**

A National Health Insurance pilot district compared to a non-pilot district.

**Methods:**

A quasi-experimental ecological study design was used to compare selected PHC utilisation indicators in the District Health Management Information System from June 2010 to May 2014 between a pilot and a non-pilot district. Both single and controlled interrupted time series analyses were used for comparing before and after implementation of the intervention.

**Findings:**

Single interrupted time series analysis showed an increase in adults remaining on anti-retroviral therapy, clients seen by a nurse practitioner and clients 5 years of age and older in both districts. However, controlled interrupted time series analysis found no difference in all parametres. Despite a decrease in total headcounts in both districts using single interrupted time series analysis, controlled interrupted time series analysis found no differences in all parameters before and after the intervention.

**Conclusions:**

The increase in utilisation of PHC services in the pilot district may not be attributable to the implementation of contracting private MPs, but likely the result of other healthcare reforms and transitions taking place in both districts around the same time.

## Introduction

Universal Health Coverage (UHC) is a pillar of sustainable development and global security.^1^ Globally, it is estimated that 400 million people lack access to essential health services, and 150 million suffer financial catastrophe because of out-of-pocket (OPP) expenditure on health services.^2^ Despite accounting for 25% of the global burden of disease, sub-Saharan Africa is home to approximately 3% of the world’s medical practitioners (MPs).^3^ To correct this, 1 million new MPs will need to be trained and deployed in the next 10 years.^4^ Thus, the World Health Organisation (WHO) recognises the attainment of UHC as a goal for 2030 for all member countries.^5^ The UHC goal seeks to address healthcare access, utilisation and quality challenges facing public healthcare.^6,7^

Progress towards UHC in South Africa has been hampered by inequitable distribution of MPs between the two-tiered system of public and private providers, which needs to be addressed.^8^ The South African public health sector is staffed by 30% of MPs to service the 70% of the uninsured population. The remaining 70% of MPs cater for 15% of the population who have medical insurance.^9^ Perceived inferior services in the public compared with the private healthcare system mean that 25% of the uninsured population pay OPP for private-sector care.^10,11^ Thus, in 2012, the South African government, through the National Department of Health (NDoH), launched the National Health Insurance (NHI) pilot project and Human Resources for Health (HRH). strategy as part of a plan towards addressing the inequitable distribution of MPs.^12^ The first phase of NHI focusing on primary healthcare (PHC) was implemented from 2012 to 2017 in 11 pilot districts. A key component of this was contracting private MPs to provide PHC services in community clinics.^13^ Nurse practitioners (NPs), and not MPs, have historically provided services at public PHC facilities. Thus, the NHI white paper recommended contracting private MPs to strengthen the public PHC system. It was envisaged to lead to an improvement in clinical quality of care, offsetting of capacity constraints, expansion of range of services, provision of clinical leadership and capacity building of the PHC team.^14,15^ The result of this would be increased utilisation of PHC services, including those of HIV services.^16^ The latter services (for HIV or AIDS) have been largely supported from 2007 to 2011 by the US President’s Emergency Plan for AIDS Relief.^17^

Health services utilisation as a surrogate measure of access is an important health outcome indicator.^18^ Effect of the NHI private MPs contracting on utilisation of PHC services is crucial for planning, appropriate allocation of scarce resources and determination of barriers to access PHC services.^19^ Absence of these data means that there is no comprehensive indicator for tracking progress and benchmarking performance of the programme. Utilisation of PHC services for planning has been monitored using routinely collected sequential district-level data on headcounts using the District Health Management Information System (DHMIS) database.^20^ However, the effects of NHI private MPs contracting pilot programme on the utilisation of PHC services have not been well explored. These data are imperative for informing legislation and policies to govern the roll out of NHI in South Africa at present. Thus, an assessment of the NHI pilot programme in attaining the intended goals is critical and hence this study. To our knowledge, this is the first study to determine population-level formative effects of UHC on PHC headcounts of adults remaining on anti-retroviral therapy (ART), clients seen by an NP, total PHC headcounts and of clients of 5 years of age and older in South Africa.

## Methods

### Research design

We adopted a quasi-experimental ecological study to investigate the causal effect of private MPs contracting on utilisation of PHC services of the NHI pilot programme in a pilot NHI district by comparing utilisation of PHC services with a non-NHI pilot district.

### Study setting

The study was conducted in one of the NHI pilot districts of South Africa, which has a population of 2 921 488 people, receiving PHC services from 68 facilities (PHC facility ratio of 1:36 980). The estimated medical scheme coverage in the district at the time of the study was 33.2%, and the Department of Health (DoH) expenditure on PHC was 56.7%.^21^ It is also the most diverse district in terms of socio-economic status of the population. The findings were compared with those in a non-NHI district with a population of 3 178 470 people accessing 90 PHC facilities (PHC facility ratio of 1:42 421). The estimated medical scheme coverage was 25.5% and DoH expenditure on PHC services was 83.1%.^21^ Selection of comparison district was not only based on proximity of the two districts but also on similarities in demographic profiles, being under the same provincial government and uniformity in the implementation of health programmes. The districts also have a similar burden of disease profile as measured by death by broad cause, namely, injuries, non-communicable diseases, HIV, TB and communicable diseases.^22^

### Study population

We studied the population of children above 5 years old and adults utilising public PHC facilities in an NHI pilot and a non-NHI district from June 2010 to May 2014. However, PHC clients utilising services less likely to be affected by the presence of MPs at the community clinics (nurse-driven services), such as maternal, child health and reproductive services, were excluded from the study.^23^

### Data collation and management

In this study, we used routinely collected secondary data. District Health Management Information System monthly reports from June 2010 to May 2014 for the two districts were collated. Each PHC facility in the district collected data and sent them to a DHMIS officer, who created electronic formats (in Microsoft Excel). Data in DHMIS were deemed complete for the selected variables as the values for the elements were reported monthly for the period of the study. We used PHC headcounts because PHC is the focus of the implementation of the first phase of the NHI pilot programme. The complete list of variables, definitions, use, impact model and mechanism of the impact model is shown in Table 1.

**TABLE 1:** Definitions and impact model of primary healthcare data elements and indicators in District Health Management Information System.

Data element	Definition	Use and context	Proposed impact model	Mechanism of impact model
Adult remaining on ART at the end of the reporting period	Total number of adults remaining on treatment for HIV at the end of the reporting month	Used in calculating ART client remaining on ART at the end of the month, which monitors the total adults remaining on lifelong ART at the end of the month	Level and slope change	Improvement in service delivery would lead to increase in number of patients willing to be seen at PHC facilities
PHC client seen by a nurse practitioner	A PHC client of any age consulted or treated by a professional nurse for a PHC service	-	Slope change	Contracting of MPs to provide services at PHC facilities would reduce the load on professional nurses who have been providing services in these facilities
PHC headcount of clients 5 years of age and older	All individual clients of 5 years of age and older seen for PHC	Monitors PHC access and utilisation by clients 5 years of age and older	Level and slope change	Improvement in service delivery would lead to increased confidence in the PHC facilities and privately contracted MPs spend more than 90% of their time on those in this age
Total PHC headcount	All individual clients seen for PHC. Clients of all ages attending the facility for PHC. Each client is counted once a day regardless of the number of services provided on that day.	-	Level and slope change	Improvement in service delivery at PHC facilities, envisaged from contracting of private MPs, would significantly increase total PHC headcounts
PHC client seen by private doctor	A PHC client of any age consulted or treated by a private doctor on contract for a PHC curative and preventative service. The service is provided in a public health facility.	Monitoring of services rendered by private doctors employed on contract to consult PHC clients in public health facilities in accordance with the NHI objectives to increase doctor coverage	Level and slope change	Contracting of MPs to provide services at PHC facilities would lead to increase in the number of patients seen by this category of staff
PHC client seen by public doctor	A PHC client of any age consulted or treated by a doctor employed in the public sector for a PHC curative and preventative service	Curative services entail the diagnosis or treatment of clients. This data element should be collected in all PHC facilities with full- or part-time doctors. Clients might originally be seen by a professional nurse for a PHC service or may be seen directly by the doctor.	Slope change	Contracting of MPs to provide services at PHC facilities would reduce the load on public doctors who have been providing services in these facilities

PHC, primary healthcare; ART, anti-retroviral therapy; MP, medical practitioner.

The use of a comparison district was done to control for time varying confounders. Interrupted time series analysis (ITSA) compared selected PHC data elements across time within the single population of an NHI pilot district accounting for underlying trends in the outcomes, which avoids between-group differences such as selection bias of unmeasured confounders. However, this did not exclude confounders, which do not form part of the underlying trend, such as interventions or events occurring around the time of the NHI pilot project. To limit these threats, we selected a control district of a non-NHI pilot district to control for other examples of time varying co-interventions implemented in both districts that could affect the outcomes.

The selection of variables and time points was based on requirements of analysis using both single and controlled ITSA.^24^ We measured and compared selected monthly PHC headcounts in the two districts, which met the criteria of ability to change relatively quickly after the implementation of MPs contracting or after a clearly defined lag.^24,25^ The unit measure for selected data points was months as per DHMIS reporting. A total of 48 time periods, with 24 before (June 2010 to May 2012) and 24 after (June 2012 to May 2014) implementation of contracting MPs, were selected. The minimum required for ITSA is 10 before and 10 after implementation of a programme to have at least 80% power. The selection can detect a change level of at least 5 standard deviations of the pre-data if the autocorrelation is > 0.4.^26^

## Statistical analysis

Firstly, we did a single-group ITSA of selected PHC secondary data elements from the DHMIS,^27,28^ in which the pre-intervention trend was projected for the post-intervention to serve as a counterfactual for each district separately. We estimated regression coefficients by the ordinary least squares’ method, producing Newey–West standard errors. Regression diagnostics were tested using the Cumby–Huizinga test on the error distribution in order to test for residual autocorrelation, which was adjusted for by Prais–Winsten regression. This was carried out to determine whether there was a statistically significant difference between the pre- and post-intervention slopes outside the intervention period in both districts. The series of monthly counts were assumed to follow a Poisson distribution allowing for overdispersion. Seasonality was accounted for using dummy variables in order to define each month of the year; dummy variables were being used as adjustment factors in the analysis.

The formula used for this ITSA was:

$$Y_{t}=\beta_{0}+\beta_{1}T_{t}+\beta_{2}T_{t}+\beta_{3}X_{t}T_{t}+e_{t},$$ [Eqn 1]

where *Y_t_* is the aggregate outcome variable measured at ‘t’ equally spaced time points; *T_t_* is time elapsed since the beginning of the study; *X_t_* (indicator) represents the intervention; *X_t_T_t_* is an interaction term; *e*_*t*_ is the random error; *β*_0_ is the intercept of the line on the vertical axis (initial level of the outcome variable); *β*_1_ is slope or trajectory of the outcome variable before the intervention; *β*_2_ is change in level of outcome immediately after the introduction of the intervention and *β*_3_ is the difference between the pre- and post-intervention slopes. Therefore, significant values of *p* at *β*_2_ indicate an immediate intervention effect, and significant values of *p* at *β*_3_ indicate an intervention effect over time.

We then used the Prais–Winsten AR (1) model of regression to perform a multiple-group ITSA or controlled interrupted time series analysis (CITSA). This allowed us to control for both the pre-intervention trends in the outcomes and the potential confounding events that would have affected both the control and the study groups. A Poisson distribution was assumed, as all the outcomes used in the study were individual counts. We modelled the association as a slope change rather than an immediate level change because the selected outcomes were likely to change gradually as MPs were contracted. Multiple-group ITSA was performed according to Linden’s description.^29^ We estimated differences in coefficients according to the following formula:

$$Y_{t}=\beta_{0}+\beta_{1}T+\beta_{2}X_{t}+\beta_{3}TX_{t}+\beta_{4}Z+\beta_{5}ZT+\beta_{6}ZX_{t}+\beta_{7}ZTX_{t}+e_{t},$$ [Eqn 2]

where *Z* is a dummy variable for assignment to treatment or control.

Stata statistical software package, version 16 (StataCorp, USA), was used to perform all the statistical analyses.

### Ethical consideration

Ethics approval for this study was obtained from the University of Witwatersrand Human Research Ethics Committee (HREC) (Certificate number: M180956). The DHMIS data are aggregated at district level; thus, it does not contain any identifiers.

## Results

### Population characteristics

Table 2 shows that the population increase in the non-NHI pilot and NHI pilot district in the period 2010–2014 was similar at 2.5% and 3%, respectively, with similar proportions for different age groups. However, over the same period, the pilot district had a higher percentage of the population able to access medical care from the private healthcare providers.^30^ Conversely, the non-pilot district had a lesser PHC clinic provider-to-population ratio. Over the same period in both districts, there was an increase in the selected PHC headcounts.

**TABLE 2:** Population and primary healthcare characteristics of the pilot and non-pilot districts.

Variable	NHI pilot district					Non-pilot district				
	2010	2011	2012	2013	2014	2010	2011	2012	2013	2014
**Population**^32,33^	2 836 329	2 921 488	3 012 054	3 105 428	3 201 696	3 091 226	3 178 470	3 256 978	3 337 426	3 419 860
**Age**										
Young (1–14 years)	24.6	24.6	23.2	23.2	23.2	24.6	24.6	24.3	24.3	24.3
Working age (15–64 years)	71.9	71.9	71.9	71.9	71.9	71.7	71.7	71.7	71.7	71.7
Elderly (≥ 65 years)	4.4	4.4	4.9	4.9	4.9	3.5	3.5	4	4	4
**Percentage with medical insurance**^34^	33.2	33.2	33.2	33.2	33.2	25.5	25.5	25.5	25.5	25.5
**PHC data elements**										
Mean adults remaining on ART at the end of the month	23 896	36 971	73 137	104 411	119 549	14 785	46 370	107 666	139 897	156 401
Mean PHC client seen by a nurse practitioner	2 199 849	3 707 856	4 235 461	4 035 493	1 625 503	2 391 364	4 510 063	5 337 516	5 368 390	2 198 738
Mean PHC total headcount	2 240 454	4 551 161	4 951 020	4 951 461	2 002 915	2 809 489	5 313 134	6 096 612	6 159 529	2 532 644
Mean PHC headcount of 5 years of age and older	2 111 853	4 037 369	4 023 867	4 051 447	1 663 298	2 232 836	4 250 244	4 942 652	5 005 505	2 055 648

PHC, primary healthcare; ART, anti-retroviral therapy.

### Time series plots

**FIGURE 1:**
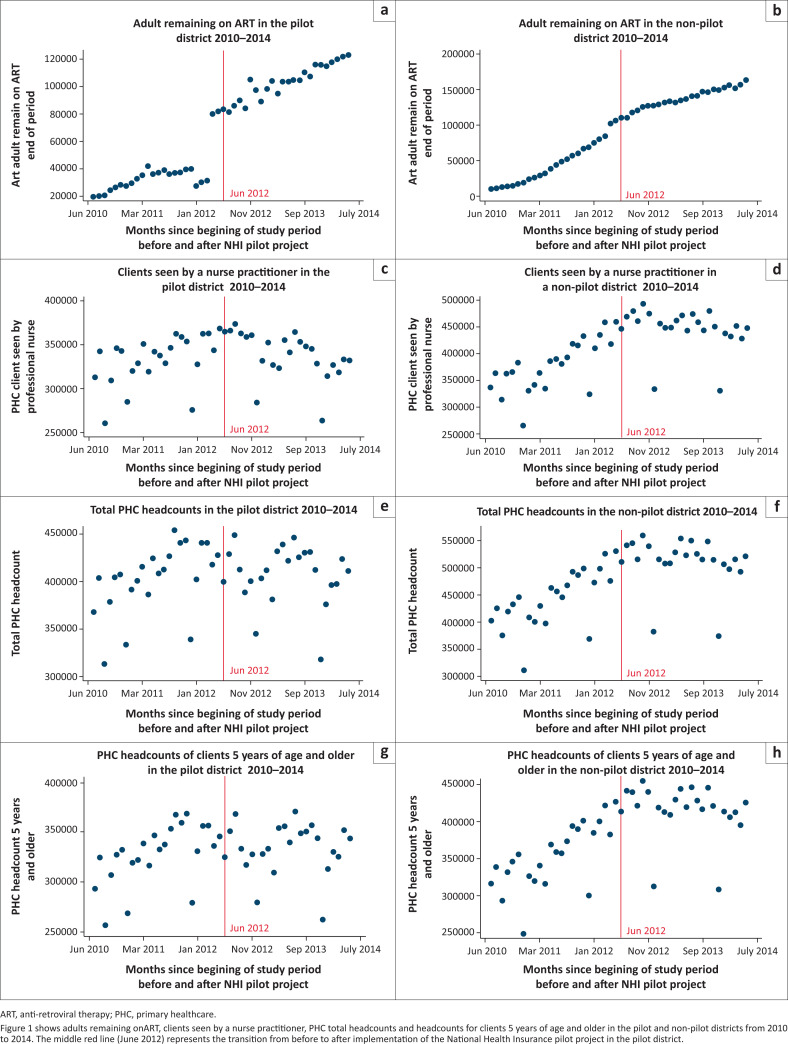
Graphical representation of selected primary healthcare utilisation indicators.

Figure 1 shows time series plots of the selected PHC data elements in both districts from 2010 to 2014. There was a more pronounced increase in adults remaining on ART in the pilot district than the non-pilot district. Conversely, there was a higher increase in PHC headcounts for clients of 5 years of age and older, total PHC headcounts and clients seen by an NP in non-pilot district than a pilot district.

The intersection for NHI MPs contracting assumed a 3-month lag period for recruitment processes after March 2012, which was when an announcement to start NHI activities was made.^31^ A 12-month progress report in 2013 showed that contracting of MPs for PHC facilities was more than 75% complete in May 2012 – July 2012 in all pilot districts.^32^

### Interrupted time series analysis and controlled interrupted time series analysis

**FIGURE 2:**
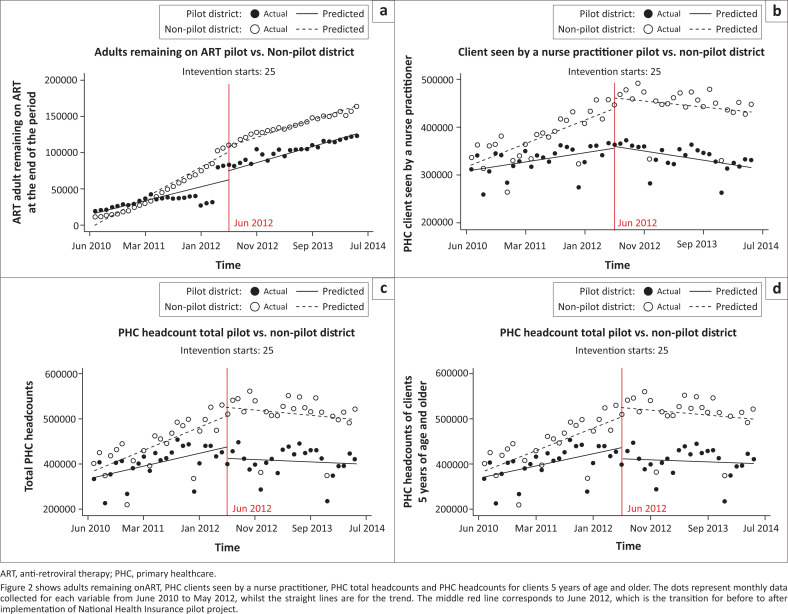
Controlled interrupted time series regressions for selected primary healthcare utilisation indicators.

Table 3 shows pre- and post-intervention headcounts of adults remaining on ART in each district, separately using ITSA. In both districts, there was an increase in adults remaining on ART, but it was higher in the pilot district than in a non-pilot district (relative risk [RR], 1.65; 95% confidence interval [CI], 1.64–1.66; *p* < 0.0001; vs. RR, 1.32; 95% CI, 1.32–1.33; *p* < 0.0001). A comparison of adults remaining on ART using CITSA in Figure 2 and Table 4 shows that there was a statistically significant difference in the base level (coefficient = 16 043; 95% CI 952–31 134; *p* = 0.037) and base trend (coefficient = −2262; 95% CI, −3919 to −606; *p* = 0.008). However, there was no difference in change in level and change in trend.

**TABLE 3:** Interrupted time series analysis summary statistics.

DHMIS data element (variable)	Pilot district	Non-pilot district
**Adults remaining on ART**		
Change	Increase	Increase
Magnitude	65%	32%
RR	1.65	1.32
95% CI	1.64–1.66	1.32–1.33
*p*	< 0.001	< 0.001
**PHC clients seen by a nurse practitioner**		
Change	Increase	Increase
Magnitude	2%	6%
RR	1.02	1.06
95% CI	1.01–1.02	1.06–1.07
*p*	< 0.001	< 0.001
**PHC headcounts of clients 5 years of age and older**		
Change	Decrease	Increase
Magnitude	2%	2%
RR	0.98	1.02
95% CI	0.97–0.98	1.01–1.02
*p*	< 0.001	< 0.001
**Total PHC headcounts**		
Change	Decrease	Increase
Magnitude	2%	5%
RR	0.98	1.05
95% CI	0.97–0.98	1.04–1.06
*p*	< 0.001	< 0.001

PHC, primary healthcare; ART, anti-retroviral therapy; DHMIS, District Health Management Information System; CI, confidence intervals; RR, relative risk.

**TABLE 4:** Controlled interrupted time series analysis summary statistics.

Parameter	Coefficient (95% CI)	Standard error	*p*
**ART adults remaining on ART at the end of the period†**			
Difference: Initial mean level (base level) (β_4_)	16 043 (952, 31 134)	7593	0.037
Difference: Mean baseline slope (base trend) (β_5_)	-	834	0.008
Difference: Pre- and post-trend (change in level) (β_6_)	2065 (-175, 4306)	1127	0.07
Difference: Trend post-intervention (change in trend) (β_7_)	−197 (-1 020, 627)	415	0.636
*Durbin–Watson statistic* (*original*) = *1.2, Durbin–Watson statistic* (*transformed*) = *1.8*			
**PHC client seen by a nurse practitioner**‡			
Difference: Initial mean level (base level) (β_4_)	−10 873 (-44 831, 23 085)	17 087	0.526
Difference: Mean baseline slope (base trend) (β_5_)	−2994 (-5450, -539)	1235	0.017
Difference: Pre- and post-trend (change in level) (β_6_)	2483 (-849, 5816)	1677	0.142
Difference: Trend post-intervention (change in trend) (β_7_)	−511 (-2770, 1750)	1138	0.654
*Durbin–Watson statistic* (*original*) = *2.0, Durbin–Watson statistic* (*transformed*) = *2.0*			
**Total PHC headcount§**			
Difference: Initial mean level (base level) (β_4_)	−13 300 (-52 220, 25 618)	19 584	0.499
Difference: baseline slope (base trend) (β_5_)	−2273 (-5160, 614)	1453	0.121
Difference: Pre- and post-trend (change in level) (β_6_)	3004 (-1048, 7055)	2039	0.144
Difference: Trend post-intervention (change in trend) (β_7_)	731 (-2100, 3566)	1426	0.61
*Durbin–Watson statistic* (*original*) = *2.0, Durbin–Watson statistic* (*transformed*) = *2.0*			
**PHC headcount of clients 5 years of age and older¶**			
Difference: Initial mean level (base level) (β_4_)	−3226 (-33 544, 27 092)	15 256	0.833
Difference: Mean baseline slope (base trend) (β_5_)	−1985 (-4216, 245)	1122	0.08
Difference: Pre- and post-trend (change in level) (β_6_)	2954 (-207, 6116)	1591	0.067
Difference: Trend post-intervention (change in trend) (β_7_)	969 (-1282, 3220)	1132	0.395
*Durbin–Watson statistic* (*original*) = 2.0, *Durbin–Watson statistic* (*transformed*) = 2.0			

CI, confidence intervals; β, difference; PHC, primary healthcare; ART, anti-retroviral therapy.

† , Total number of adults remaining on treatment for HIV at the end of the reporting month.

‡ , PHC client of any age consulted or treated by a professional nurse for a PHC.

§ , All individual clients seen for PHC. Clients of all ages attending the facility for PHC.

¶ , All individual clients 5 years of age and older seen for PHC.

Table 3 shows that in the ITSA, the increase in PHC clients seen by NPs in both districts was minimal (RR, 1.02; 95% CI, 1.01–1.02; *p* < 0.001; vs. RR, 1.06; 95% CI, 1.06–1.07; *p* < 0.001). Figure 2 and Table 4 show that in the CITSA there was a statistically significant difference in base trend between the two districts (Coefficient, −2994; 95% CI, −5450 to −539; *p* = 0.017). Conversely, there was no statistically significant difference in base level, change in level and change in trend.

The ITSA in Table 3 shows that there was a marginal decrease in PHC headcounts for clients 5 years of age and older in the pilot district (RR, 0.98; 95% CI, 0.97–0.98; *p* < 0.001). However, in the non-pilot district, there was a marginal increase (RR, 1.01; 95% CI, 1.01–1.02; *p* < 0.001). Similarly, for total PHC headcounts shown in Table 3, there was a decrease in the pilot district (RR, 0.98; 95% CI, 0.97–0.98; *p* < 0.001) but an increase in the non-pilot (RR, 1.05; 95% CI, 1.04–1.06; *p* < 0.001). The CITSA for both data elements in Figure 2 and Table 4 shows that there was no statistically significant difference in base level, base trend, change in level and change in trend.

## Discussion

Our findings using ITSA showed 65% and 32% increase in the number of adults remaining on ART in the pilot and non-pilot districts, respectively. However, CITSA found a difference in base level and base trend but not in post-intervention parameters. Both districts showed an increase in clients seen by an NP using ITSA of 2% for pilot and 6% for non-pilot, but only a difference in base trend using CITSA. Interrupted time series analysis of total headcounts and headcounts of 5 years of age and older clients showed a 2% decrease in both districts for the former, but an increase of 1% and 5% in the pilot and non-pilot districts, respectively, for the latter. Controlled interrupted time series analysis found no difference in all parameters before and after the intervention. These findings are suggestive of an overall improvement in PHC indicators, except utilisation over the study period, which is not attributable to the implementation of the contracting of MPs for the NHI pilot programme.

A comparison of adults remaining on ART between the two districts using ITSA showed a differential increase of 65% for the pilot district and 32% for a non-pilot district. An increase for the earlier analysed using ITSA may suggest a causal effect of MPs contracting in the pilot district, which is in line with findings of an impact study on NHI pilot districts comparing data from 4 months prior to placing private MPs and the last 4 months of their placement.^33^ Similarly, findings of increased utilisation of PHC services post-implementation of a UHC programme were observed in Burkina Faso,^34^ Ghana,^35^ China,^36^ Mexico^37^ and Rwanda.^38^ However, comparison using CITSA found that, despite prior difference in base level and trend, after the intervention there was no change in all parameters. Given that the project was only implemented in the pilot district, this finding suggests that the outcome could be attributable to an intervention common to both districts. Thus, the increase in the number of adults remaining on ART in the pilot district cannot entirely be attributable to the implementation of the MPs contracting in the NHI pilot project. It may be attributed to the roll out of HIV testing and ART in all districts and the morbidity transition in South Africa, which increased from 2010 and peaked in 2012.^39^ This is also in line with the HIV treatment programme milestones, such as increased testing and ART initiation as a result of NDoH significant increase in resource allocation and peak of US President’s Emergency Plan for AIDS Relief support in 2012.^17,40^ Differential findings between ITSA and CITSA, attributable to different interventions aside from MPs contracting of the NHI pilot programme, illustrate the strength of our approach in this study.

As core providers of services in PHC facilities, it was important to determine the effect of private MPs contracting on the headcounts of clients seen by an NP.^41^ They provide PHC services such as reproductive, maternal and child health. Comparison of pre- to post-intervention independently using ITSA showed increases of 2% and 6% in pilot district and non-pilot district, respectively. This could potentially lead to a compromise of quality of PHC services because of increased workload for the NPs.^42^ However, the predicted plots, given the post-intervention trends, show that after the intervention there will be a predicted decline in both districts. This is a positive finding in the sense that excessive workload on NPs has been associated with dissatisfaction with work, burnout and stress.^43,44,45^ Conversely, on comparing the two districts using CITSA, the only difference was in base trend and not in any other measured parameters. This may be because of a confounder that was implemented in both districts such as other aspects of PHC re-engineering, namely, establishment of municipal ward-based PHC outreach teams, integrated school health programm and district clinical specialist teams in all districts.^41,46^

The total PHC headcounts were found to be equivalent to the sum of headcounts for clients seen by an NP and by public MPs before the NHI pilot project and after, and to headcounts of clients seen by an NP, public MPs and private MPs. On comparison of the total PHC headcounts and those of clients 5 years of age and above using ITSA, an increase of 2% and 5% was observed in the pilot and non-pilot districts, respectively. This could be explained by in-migration, which over the period of the study was found to be higher in the non-pilot than in the pilot district.^47^ However, in the pilot district, there was a 2% decrease in both data elements, a reduction in utilisation of PHC services after the introduction of NHI pilot project, which is in contrast to findings in other developing countries.^48,49,50^ This could be explained by the difference in the contracting models between South Africa and other countries, where health insurance was extended to underserved communities and healthcare providers compensated by the insurance fund for their services. However, the contracting model for MPs implemented in the first phase of NHI pilot districts in South Africa was, for contracted MPs, to be physically placed at PHC facilities and compensated by way of a salary irrespective of number of patients seen. Nevertheless, there was no difference in all the parameters of CITSA.

In all the measured variables using CITSA, it was clear that after the implementation of NHI pilot project, there was no difference in parameters when compared with non-pilot districts. However, in the ITSA, there was an improvement in the selected data elements. These findings show that in as much as there was no effect attributable to implementation of the MPs contracting of the NHI pilot project in pilot districts, there were positive findings in both districts. These findings may be attributable to implementation of programmes common to both districts. Before adding MPs contracting in the implementation of the NHI pilot project as the fourth stream of PHC re-engineering in 2012, the NDoH had been implementing three other streams at PHC level since 2011 in all districts.^46^ It may be that these had a positive effect on PHC in the districts.

Effects of contracting private providers on utilisation of PHC services in the context of our study sites may also have been influenced by other confounders such as socio-demographic factors and other health reforms implemented around the same time.^51^ These include population density, access to PHC facilities, quality of care and epidemiological transition.^52^ From 2010 to 2015, HIV became the biggest contributor to the burden of disease in South Africa as testing and treatment became readily available, resulting in increased demand for health services because of scale-up of HIV treatment.^39^ However, studies informing decisions to implement UHC did not take this into consideration.^53^

Despite the existence of an association between perceived quality of healthcare and utilisation,^50^ our study inferences were limited by lack of a quality measure. However, a postulation can be made to explain the findings. Of the four outcomes under investigation, only the number of people remaining on ART, when comparing pre- to post-MPs contracting, showed a significant increase in both districts, but the others reduced or minimally increased. This could be explained by the fact that an increase in provision of ART has significantly reduced morbidity in the districts to an extent where utilisation for other HIV-related health services has reduced.^41^ Thus, we can hypothesise that providing ART to all HIV-positive people will lead to a reduction in utilisation of PHC services and workload. However, inferences of the perceived quality of care at the PHC level can be made from the utilisation of these services at the higher levels of care.^50^

There are several limitations to the interpretation of our findings. Firstly, the lack of effect attributable to MPs contracting in the selected PHC data elements in the pilot district may be because of limited number of contracted MPs and, in some instances, the limited time they spend at the clinics.^14^ By the end of 2014, there were only 77 MPs in the district working in only 17 out of 68 PHC clinics.^54^ However, an NHI report for the same period showed that contracting of MPs was more than 75% complete by the end of 2012.^23^ Secondly, the presence of a statistically significant difference in base level and base trend in the CITSA comparing pilot with non-pilot districts in adults remaining on ART and difference in base trend in PHC clients seen by an NP shows that our selection of a control district based on a closely matched region having similar baseline characteristics may have limited the validity of the study. However, selection of control based on location was the only possible method as NHI pilot project is a community intervention. Thirdly, the close proximity of the two districts may have made the findings subject to contamination, substitution and migration effects.^24,47,55^ Finally, use of secondary data (DHMIS) that is collected in a non-research setting may not be powered to detect differences between groups.^56^ However, these data are subject to both random and systematic methods, which were countered by the research methodology and analytical methods employed in the study. Our interpretation of findings also took note of any changes in the way data were measured or collected, which may have taken place during the study and could limit inferences. To mitigate these limitations, we applied robust statistical methods during the analysis.

## Conclusion

Our findings suggest that the implementation of private MPs contracting of the NHI pilot project may not have resulted in the observed changes in PHC indicators. Rather, co-interventions implemented in all districts such as PHC re-engineering and other HIV programmatic shifts may have done so.

### Recommendations

An understanding of the reasons behind our findings would be made by determining the perceived quality of care at the PHC level in the districts as these have an effect on utilisation of services. Because quality of care at the PHC level was beyond the scope of this study, we recommend that further studies be done to understand the quality of care at PHC level, both perceived by patients and clinically.
